# Global and gene-specific DNA methylation in adult type 2 diabetic individuals: a protocol for a systematic review

**DOI:** 10.1186/s13643-018-0708-7

**Published:** 2018-03-15

**Authors:** Tinashe Mutize, Zibusiso Mkandla, Bongani B. Nkambule

**Affiliations:** School of Laboratory Medicine and Medical Sciences (SLMMS), College of Health Sciences, University of KwaZulu-Natal, Durban, 4001 South Africa

**Keywords:** Global DNA methylation, Gene-specific DNA methylation, Genome-wide DNA methylation, Epigenetics, Type 2 diabetes mellitus

## Abstract

**Background:**

DNA methylation (global and gene-specific) has been reported as an epigenetic mechanism that could be involved in the pathogenesis of type 2 diabetes mellitus (T2DM). Furthermore, epigenetic therapy has been suggested as a future possibility for T2DM treatment. Epigenetic changes illustrate the environmental link of the disease. Since some of the epigenetic modifications can be reversed, they could be used as potential therapeutic targets. The aim of the systematic review will be to synthesise the available evidence pertaining to the link between DNA methylation and T2DM. The systematic review will evaluate characteristics of reported studies such as the source of DNA used, methods of quantifying DNA methylation and the participants’ demographics (age, gender, race and adiposity). We will conduct a narrative synthesis of data, and if there are an adequate number of sufficiently homogenous studies, we will consider performing a meta-analysis. The review will evaluate if the levels of DNA methylation are a possible risk factor for T2DM. Furthermore, we will assess whether DNA methylation is a plausible biomarker and therapeutic target for the treatment and management of T2DM.

**Methods:**

This systematic review protocol will be reported in accordance with the Preferred Reporting Items for Systematic Reviews and Meta-Analyses Protocols (PRISMA-P) 2015 statement. An extensive search for original research articles, published since inception, was performed on major databases such as Embase, MEDLINE and Cochrane Library. The search strategy will include a combination of key words and MeSH words. Literature that is available in English and studies in other languages that can be translated into English will be used. Data extraction will be done in duplicate, and two authors will independently screen for eligible studies using pre-defined criteria. The Cochrane Risk of Bias Assessment Tool and Joanna Briggs Institute (JBI) Critical Appraisal tools will be used to assess the risk of bias. The Grading of Recommendations, Assessment, Development and Evaluation assessment tool will be used to assess the overall quality of extracted data.

**Discussion:**

This systematic review will evaluate published literature, assessing the link between DNA methylation and T2DM. Our findings could help guide future research evaluating epigenetic changes in T2DM and direct future therapeutic interventions.

**Electronic supplementary material:**

The online version of this article (10.1186/s13643-018-0708-7) contains supplementary material, which is available to authorized users.

## Background

Type 2 diabetes mellitus (T2DM) is a complex and multifactorial metabolic disorder caused by genetic and environmental factors [1]. It is a major source of morbidity and mortality worldwide [2]. The identification of individuals who are at risk of developing T2DM could facilitate early intervention strategies to delay or prevent their progression to disease, therefore resulting in the minimisation of disease burden [3].

Epigenetic mechanisms are heritable changes in the genome which are not affected by a change in the nucleotide sequence [4]. These include histone methylation, lysine methylation, histone phosphorylation, RNA interference (RNAi) and genomic imprinting [5–7]. DNA methylation is a widely studied epigenetic mechanism, which offers a unique opportunity for the identification of potential biomarkers for an increased risk of developing T2DM. Gene-specific DNA methylation refers to the analysis of the methylation status of specific genes whereas global DNA methylation refers to the average methylation status that occurs across the whole genome [8].

DNA methylation is a normal physiological process involved in gene expression control and, therefore, could result in disease when it happens in a wrong way [9]. Aberrant DNA methylation could be one of the pathogenic factors involved in the initiation and progression of T2DM [10, 11]. DNA methylation refers to the covalent modification when a methyl group is added to hydrogen on position 5 of cytosine nucleotides (H5), primarily in CpG islands in the promoter regions of genes. This process is catalysed by the DNA methyltransferase (DNMT) enzymes, with *S*-adenosyl-methionine as the methyl donor [12, 13]. In fact, aberrant DNA methylation has been associated with the pathogenesis of T2DM and has been reported to be observable during the subclinical or asymptomatic stage of the disease [14, 15]. Furthermore, the possibility of DNA methylation acting as a potential biomarker for metabolic diseases such as T2DM and cardiovascular disease has been reported [14, 16–18].

Generally, DNA hypermethylation is believed to cause gene silencing whereas DNA hypomethylation has been associated with gene activation [10]. Several studies have reported the link between diabetes mellitus and DNA methylation (gene-specific as well as global). For global DNA methylation, some studies reported hypomethylation [19–21] and some reported hypermethylation [17, 22]. For gene-specific DNA methylation, some studies reported hypermethylation [10, 23–25] and another study reported hypomethylation [26].

The difference in DNA methylation trends (hypo- or hypermethylation) could be due to the different study populations, techniques used or different conditions of the diabetes mellitus and metabolic syndrome parameters in the studies. Therefore, these discrepancies warrant doing a systematic review in the light of the capability of DNA methylation as a possible biomarker, risk factor and prognostic marker for T2DM and the other metabolic syndrome conditions. Moreover, DNA methylation is reversible, thus enabling intervention strategies to possibly reverse the disease phenotype and subsequently reduce the micro- and macrovascular complications associated with the disease.

### Objectives

This systematic review aims to assess the results of published data and summarise the knowledge base on the link between DNA methylation and T2DM. This study aims to evaluate the link between DNA methylation profiles (global and gene-specific) of T2DM individuals compared to their healthy counterparts. In addition, this study will also assess the possibility of DNA methylation as a high-risk biomarker and intervention targets for the disease.

## Methods

This systematic review protocol will be reported in accordance with the Preferred Reporting Items for Systematic Review and Meta-Analyses Protocols (PRISMA-P) 2015 statement [27]. A populated checklist for this review protocol has been provided as PRISMA-P checklist docx (Additional file 1).

### Protocol and registration

This systematic review protocol was submitted on PROSPERO for registration.

### Eligibility criteria (inclusion and exclusion criteria)

The study will include all research studies with available full texts published since inception. All articles published in English and those translatable using Google Translate will be considered. Only studies using human subjects (DNA from humans) will be considered. This study will include both interventional and observational studies, inclusive of randomised controlled trials, cohort studies, case-control studies, as well as cross-sectional studies. In addition, studies looking at gene-specific methylation as well as global DNA methylation will be considered.

### Information sources

Studies published in Embase, MEDLINE and Cochrane Library will be used as the literature sources. For unclear or unreported parameters in the studies such as the number of participants, efforts will be made to contact the authors and obtain the missing information.

### Search strategy

The search strategy will be developed using medical subheadings (MeSH) and key words related to type 2 diabetes mellitus and DNA methylation. The key words and MeSH terms will include hyperglycaemia, epigenetics, global DNA methylation, gene-specific DNA methylation and genome-wide DNA methylation. In order to eliminate any discrepancies and inconsistencies regarding reviewers’ inclusion and exclusion of studies, a structured search involving two independent reviewers (TM and ZM) will be employed when identifying study titles and abstracts. Electronic bibliographic databases that will be searched will include MEDLINE, Embase and Cochrane Central Register of Controlled Trials.

A MEDLINE search strategy will be developed by the project team and then peer reviewed by the Health Sciences Librarian with experience in systematic review searching. The MEDLINE search strategy will then be adapted for other databases. Each database will be searched using the same search strategy adjusted for the database syntax, so as to eliminate any inconsistencies that may affect data extraction. A complete search strategy using MEDLINE is shown on the attached documents, namely Search Strategy docx (Additional file 2). The database search will be supplemented by scanning the reference lists of included studies to identify relevant studies.

### Study selection

Literature from the databases will be screened using an appraisal worksheet (STROBE). The appraisal worksheet will contain subheadings such as aims and objectives of the study, source and origin of DNA used, limitations of the study, year published, sample size, techniques used to assess DNA methylation, statistical methods used, confounding factors, cross-sectional or longitudinal study and if participants were male or female. The Mendeley reference manager will be used to detect and remove duplicates. The appraisal of studies will be documented using Microsoft Excel. In order to eliminate any discrepancies and inconsistencies regarding reviewers’ inclusion and exclusion of studies, a structured search involving two independent reviewers (TM and ZM) will be employed when identifying study titles and abstracts.

### Data collection process

In order to ensure that relevant data for this review is collected, a structured form containing the following information will be created: first author’s details (name and year of publication), author’s country, study type used, sample size, age and gender of participants, weight status, assays and techniques performed, as well as the statistical method used in the analysis. Extracted studies will be carefully assessed by two different authors (TM and ZM) of this review so as to remove any duplicates that may exist. In case of disagreements, one of the authors (BBN) will adjudicate.

### Data items

Data item classification will be listed and defined using the PECO (population, exposure, controls and outcomes) method. The population would be adult T2DM human participants of any race. The exposure will be the hyperglycaemia and the DNA hyper- or hypomethylation status of the participants. The comparator will be normoglycaemic individuals. The primary outcome would be to assess the DNA methylation profile of type 2 diabetic individuals. The secondary outcome would be to assess the DNA methylation profile results in different metabolic syndrome parameters or risk factors such as obesity and cardiovascular disease (CVD).

### Data simplification

Studies that mention that participants were on diabetic treatment (such as metformin) and those who were not on diabetic treatment will be grouped as the treatment group and the non-treatment group as a data simplification measure.

### Risk of bias in individual studies

The Cochrane Risk of Bias Assessment Tool will be used to assess bias [28]. This tool encompasses various domains which are used to detect bias in reporting. The Grading of Recommendations, Assessment, Development and Evaluation (GRADE) assessment tool will be used to assess the overall quality of extracted data [29]. The Joanna Briggs Institute (JBI) Critical Appraisal tools with specific checklists for non-randomised experimental studies will be used to assess the risk of bias [30]. The JBI appraisal tools will be used to assess the methodological quality of each study through determination of the extent to which the possibility of bias in its design, conduct, as well as analysis that would have been conducted. Furthermore, this study will also discuss the exclusion of unpublished data within the final manuscript. Bias will be assessed at both the outcome and study levels.

### Qualitative synthesis and interpretation

The included studies will be described in detail and presented in a table. This systemic review is meant to be exploratory; thus, descriptive details of the methylation status association with glycaemic status and other risk factors of T2DM such as obesity, cholesterolaemia and CVD will be reported.

### Quantitative synthesis

In case of excessive heterogeneity, the results will be synthesised narratively. A transparent approach will be used to minimise the potential for bias. The *I*^2^ statistic, as a measure variance between studies, will be utilised to analyse the statistical heterogeneity between studies [31]. If the included studies are homogenous in terms of study population, design and outcomes, we will conduct a meta-analysis using inverse variance weighting to calculate a pooled effect estimate. In case-control studies, we will present pooled odds ratios (ORs), and for cohort studies, rate ratios or hazard risks (HRs). As a primary effect measure of the association between DNA methylation and diabetes, we will use the odds ratios, and as a secondary effect measure, we will use the risk ratios.

Statistical heterogeneity will be assessed using the chi-square test for homogeneity with a level of significance, alpha = 0.10 and the *I*^2^ statistic to quantify inconsistency. We will consider 50% indicating moderate or substantial heterogeneity. The variables that will be used to detect sources of heterogeneity will include study design, study population, sample size, age and glycaemic status. Meta-regression and subgroup analysis will be used to compare summary estimates from different study-level characteristics. A funnel plot will be used to investigate the risk of publication bias. In addition, Harbord’s test and Peter’s test will be used to statistically evaluate funnel plot asymmetry and the possibility of publication bias [28]. All analyses will be performed using R statistical software (The R Foundation for Statistical Computing, Vienna, Austria) and the Cochrane Review Manager 5.3 software.

### Risk of bias across studies

Risk of bias across studies which could affect cumulative evidence will be assessed by checking the limitations of the studies and if all studies had the same limitations, journals where published and if there was any evidence of selective reporting across the studies.

## Discussion

This systemic review will assess the link between DNA methylation and T2DM. The study aims to shed more light on the plausibility of the epigenetic mechanism in directing therapy, addition to other known risk factors of T2DM as well as utilisation as a biomarker of the disease. Various studies have postulated the therapeutic potential of evaluating DNA methylation may confer, but comprehensive and evidence-based reviews assessing the therapeutic potential of such treatment modalities are limited. An extensive synthesis of the available data will allow identification of evidence gaps and direct future research that will interpret the link between DNA methylation and T2DM. Furthermore, the findings of the systematic review will be disseminated through peer-reviewed publication and presented at national and international conferences.

## Additional files

Additional file 1: PRISMA-P (Preferred Reporting Items for Systematic Review and Meta-Analysis Protocols) 2015 checklist: recommended items to address in a systematic review protocol*. (DOC 82 kb)

Additional file 2: Search strategy run 12 August 2017. (DOCX 17 kb)

## Abbreviations

CVD: Cardiovascular disease
DM: Diabetes mellitus
T2DM: Type 2 diabetes mellitus
