# Supplementary material for: Global and gene-specific DNA methylation in adult type 2 diabetic individuals: a protocol for a systematic review
Source: Syst Rev. 2018 Mar 15;7:46. doi: 10.1186/s13643-018-0708-7 (PMC5856358; doi:10.1186/s13643-018-0708-7)
Supplement: Supplementary file 2 — Search strategy run 12 August 2017. (DOCX 17 kb) [file 13643_2018_708_MOESM1_ESM.docx]

**Additional file 1: Search strategy run 12 August 2017**

| **Concept 1:**  **DNA methylation** | **Synonyms to be searched (MeSH OR textwords)** | |
| --- | --- | --- |
| **PubMed** (hits=104600)  "DNA methylation" [Mesh] OR "epigenomics" [Mesh] OR ((“DNA” [Mesh] OR “Deoxyribonucleic acid“) AND (“methylation” [Mesh] OR “methylation”)) OR “DNA methylation” OR “epigenomics” OR “epigenetics” | 5-methylcytosine | 5-Methylcytosine Monohydrochloride |
|  | Epigenesis |  |
|  | Differential methylation |  |
|  | CpG methylation |  |
|  | Hypomethylation |  |
|  | Hypermethylation |  |
|  |  |  |
|  |  |  |
|  |  |  |
|  |  |  |
|  |  |  |

| **Concept 2:**  **Diabetes mellitus** | **Associated words to be searched (MeSH OR textwords)** |
| --- | --- |
| **PubMed** (hits= 423312)  ("diabetes mellitus"[MeSH Terms] OR ("diabetes"[All Fields] AND "mellitus"[All Fields]) OR "diabetes mellitus"[All Fields]) | Diabetes mellitus |
|  | Glucose metabolism disorders |
|  | Hyperglycaemia |
|  | Hyperglycemia |
|  | Metabolic diseases |
|  | Metabolic syndromes |

**Combine Concept 1 AND Concept 2** (PubMed hits=1252)
